# Characterisation of Conformational and Ligand Binding Properties of Membrane Proteins Using Synchrotron Radiation Circular Dichroism (SRCD)

**DOI:** 10.1007/978-3-319-35072-1_4

**Published:** 2016-04-28

**Authors:** Rohanah Hussain, Giuliano Siligardi

**Affiliations:** Diamond Light Source, Harwell Science and Innovation Campus, OX11 0DE Didcot, UK

**Keywords:** Circular dichroism (CD), Synchrotron radiation circular dichroism (SRCD), Protein secondary structure, Local tertiary structure, High throughput-CD (HTCD), SRCD UV-denaturation assay, Ligand binding

## Abstract

Membrane proteins are notoriously difficult to crystallise for use in X-ray crystallographic structural determination, or too complex for NMR structural studies. Circular dichroism (CD) is a fast and relatively easy spectroscopic technique to study protein conformational behaviour in solution. The advantage of synchrotron radiation circular dichroism (SRCD) measured with synchrotron beamlines compared to the CD from benchtop instruments is the extended spectral far-UV region that increases the accuracy of secondary structure estimations, in particular under high ionic strength conditions. Membrane proteins are often available in small quantities, and for this SRCD measured at the Diamond B23 beamline has successfully facilitated molecular recognition studies. This was done by probing the local tertiary structure of aromatic amino acid residues upon addition of chiral or non-chiral ligands using long pathlength cells (1–5 cm) of small volume capacity (70 μl–350 μl). In this chapter we describe the use of SRCD to qualitatively and quantitatively screen ligand binding interactions (exemplified by Sbma, Ace1 and FsrC proteins); to distinguish between functionally similar drugs that exhibit different mechanisms of action towards membrane proteins (exemplified by FsrC); and to identify suitable detergent conditions to observe membrane protein-ligand interactions using stabilised proteins (exemplified by inositol transporters) as well as the stability of membrane proteins (exemplified by GalP, Ace1). The importance of the in solution characterisation of the conformational behaviour and ligand binding properties of proteins in both far- andnear-UV regions and the use of high-throughput CD (HT-CD) using 96- and 384-well multiplates to study the folding effects in various protein crystallisation buffers are also discussed.

## Introduction

Circular Dichroism (Circular dichroism (CD))CDCircular dichroism (CD) and Synchrotron Radiation Circular dichroism (CD) (Synchrotron radiation circular dichroism (SRCD)) has been proven to be highly useful for studies of Ligand binding by soluble proteins, particularly as it is a relatively quick and easy spectroscopic measurement, requiring no extensive sample preparation, and it has the potential to be used in high throughput (HT-CD)Ligand bindingSynchrotron radiation circular dichroism (SRCD) screening (Mason 1982; Fasman 1996; Berova et al. 2000, 2012; Siligardi and Hussain 1998, 2015; Siligardi et al. 2002; Martin et al. 2011; Johnson 1990; Kelly et al. 2005; Siligardi and Hussain 2010; Jávorfi et al. 2010; Fiedler et al. 2013).

Circular dichroism (CD) spectroscopy has been widely used for the purposes of determining secondary structural content and integrity of membrane proteins (e.g. histidine kinases – Potter et al. 2002; Kim et al. 2010; Keegan et al. 2010; Yeo et al. 2008; membrane transporters – Psakis et al. 2009), protein fragments (e.g. Powl et al. 2010), and protein unfolding (Psakis et al. 2009; Keegan et al. 2010). Sreerama and Woody 2004, found that the Circular dichroism (CD) analysis of secondary structure content of membrane proteins using soluble protein reference sets was slightly inferior to that obtained for soluble proteins. The inclusion of membrane proteins in the soluble protein reference sets – now a common practise – has since improved the Circular dichroism (CD) analysis for both membrane and soluble proteins.

**Fig. 4.1:**
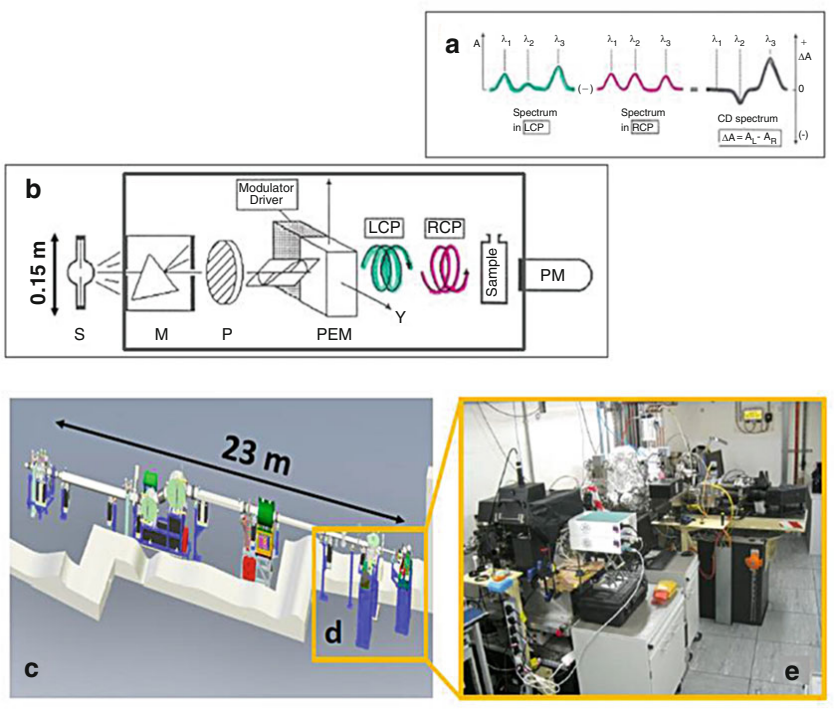
(**a**) Optical layout of a Circular dichroism (CD) spectropolarimeter: *S* light source, *M* monochromator, *P* polariser, photo elastic modulator (PEM), left circularly polarised light (LCP) LCP and right circularly polarised light (RCP), sample and detector. (**b**) Circular dichroism (CD) spectrum is the differential absorption ΔA = (A_L_ − A_R_) between LCP and RCP light. In the cartoon such a difference is greatly exaggerated. For proteins ΔA is about 10^−3^ to 10^−4^ (ΔA ≈ (measured Circular dichroism (CD) (ellipticity) in millidegrees)/3300). (**c**) The light source is a Xe-arc lamp (length =15 cm) for bench-top Circular dichroism (CD) instrument compared to the equivalent 23 m of the bending magnet (BM) of Diamond B23 beamline for synchrotron radiation Circular dichroism (CD) (Synchrotron radiation circular dichroism (SRCD)). (**d**) and (**e**) B23 has two end-stations, one dedicated for Circular dichroism (CD) imaging, cryogenic to high temperatures and HT-CD, and the other for solution and solid state (dry films) samples

In the last few decades, drug discovery has benefited from information about the three-dimensional structure of proteins at atomic resolution determined by X-ray crystallography (Carvalho et al. 2009) and Nuclear magnetic resonance (NMR) spectroscopy (Pellecchia et al. 2008). However, the number of solved human protein structures deposited in the Protein Data Bank is still only a small fraction of the approximately 20,000 protein-coding genes in the human genome (Pennisi 2012). As many proteins that cannot be studied by either X-ray crystallography due to their failure to crystallise or by Nuclear magnetic Nuclear magnetic resonance (NMR) spectroscopy (Nuclear magnetic resonance (NMR)) due to their size or irregular structure, can be characterized in terms of Protein folding by Circular dichroism (CD), which is the differential absorption between left and right circularly polarized light (Fasman 1996), see Fig. 4.1. Circular dichroism (CD) spectroscopy is sensitive to the absolute configuration and conformation of chiral molecules. With the exception of glycine, amino acids are chiral molecules adopting either L or D stereo-isomers which are non-superimposable mirror-imaged configurations. Natural proteins only consist of L amino acids.

The application of Circular dichroism (CD) spectroscopy for Ligand binding studies of membrane proteins has proved more limited, largely due to the technical challenges associated with working with hydrophobic membrane proteins. The use of the B23 beamline (Diamond Light Source synchrotron) for Synchrotron radiation circular dichroism (SRCD) has overcome the limitations encountered in studying precious membrane proteins often available in very small quantities. The B23 beamline has enabled the use of small volume capacity cuvette cells for the measurements in the Far-UV and Near-UV spectral regions that could not otherwise be used with bench-top Circular dichroism (CD) instruments. The UV protein denaturation assay, a unique feature of the B23 beamline, is another method developed to assess Ligand binding interactions, in particular for achiral fatty acid ligand analogues or small organic molecules devoid of UV chromophores, which are difficult, if not impossible to investigate with bench-top Circular dichroism (CD) instruments. The higher vacuum UV and Far-UV photon flux has proved essential for the spectroscopic characterisation of membrane proteins in high salt concentrations to enhance protein stability, which cannot be achieved satisfactorily with bench-top instruments due to absorption cut-off.

In this chapter, several examples of Synchrotron radiation circular dichroism (SRCD) spectroscopy applied to the study of membrane proteins in solution using the Diamond B23 beamline (Hussain et al. 2012a, b; Jávorfi et al. 2010) are presented. Qualitative and quantitative assessments of Ligand binding interactions of the FsrC, SbmA, and Ace1 membrane proteins, identification of functionally similar but mechanistically distinct drug targeting FsrC, and the characterisation of suitable Detergents conditions for ligand-membrane proteins Protein interactions studies are reviewed.

## Diamond B23 Beamline for Synchrotron Radiation Circular Dichroism (Synchrotron radiation circular dichroism (SRCD))SRCD

The B23 beamline is a bending magnet beamline at Diamond Light Source, UK, dedicated to Synchrotron radiation circular dichroism (SRCD)Radiation circular dichroism. The beamline consists of two distinct end-stations: module A in operation since 2011 and module B in operation since 2009, see Fig. 4.1. The beamline operates in the wavelength range of 125–650 nm, delivering a highly collimated beam with a focal spot size at the sample of 0.5 mm^2^ and a photon flux in excess of 10^12^ photons/s. The high brilliance of the beamline, coupled with its highly collimated incident micro-beam is enabling a large variety of measurements and experiments to be carried out, ranging from dilute to highly concentrated solutions, to thin solid films. The end-station of module A operates in the wavelength range of 125–500 nm and is primarily dedicated to the study of thin chiral films, whereas module B operating in the 165–650 nm is dedicated to solutions and liquid samples. A novel vertical sample chamber, which is unique among the other Synchrotron radiation circular dichroism (SRCD) beamlines around the world, has been developed to accommodate a horizontal X-Y motorised stage that can hold either the temperature controlled stage (such as Linkam™ stage), which can operate in cryogenic and high temperature conditions (from −150 °C to 300 °C), or standard microtiter or microwell plates (96- and 384-well Suprasil quartz plates (Siligardi and Hussain 2015) for high throughput Circular dichroism (CD) (HT-CD) screening.

Module B is dedicated to Ligand binding interactions that are often carried out as Circular dichroism (CD) titrations using small aperture cuvette cells that require the ability to optimise beam alignment in a fast and reproducible manner using a manual X-Y stage (Borisenko et al. 2015). This allows for measurements of dilute solutions (as low as 5 nM protein concentration) (Laera et al. 2011) using small volume capacity cells such as 0.800 ml for 10 cm pathlength or 0.020 ml for 1 cm pathlength. Synchrotron radiation circular dichroism (SRCD) stopped-flow can also be carried out in both modules. The higher photon flux of Synchrotron radiation circular dichroism (SRCD) beamlines has traditionally been considered detrimental as it promotes protein denaturation. Measures have been put in place to eliminate or greatly reduce this effect, however, this phenomenon, has also been exploited at the B23 beamline as UV protein denaturation (Hussain et al. 2012a, b; Jávorfi et al. 2010) can be conveniently used to measure accelerated protein photo stability as a function of protein formulation and qualitatively assessing and screening Ligand binding interactions (Hussain et al. 2012a, b; Longo et al. 2014, 2015). For module B, a rotating sample cell holder has also been introduced to eliminate protein denaturation induced by the higher vacuum UV and Far-UV photon flux for experiments where the UV denaturation effect is not desirable, such as thermal denaturation. Finally, to support the capabilities and potential of B23 beamline applications, a concerted software development program has been put in place. Users can now reduce and analyse their data through a single software application developed at the beamline – CDApps (Hussain et al. 2015). The software is available for users on-site, as well as via remote access, and allows novice users to quickly process their data and take full advantage of the technique.

## Ligand Binding Studies of the FsrC, Ace1 and SbmA Membrane Proteins

**Fig. 4.2:**
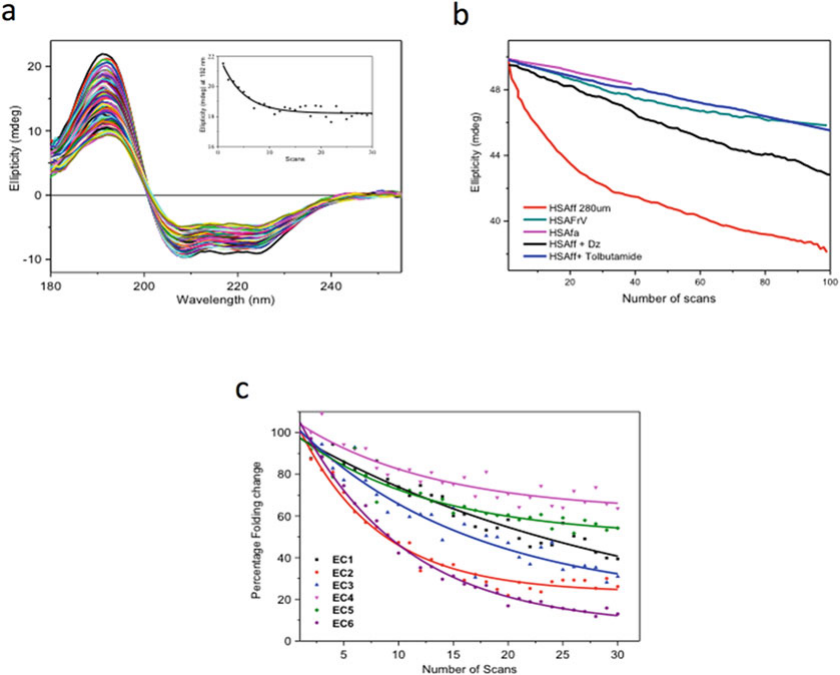
(**a**) Thirty repeated consecutive Synchrotron radiation circular dichroism (SRCD) spectra of fatty acid and immunoglobulin free human serum albumin (HSAff). The insert is the rate of protein denaturation at 190 nm. (**b**) Rate UV protein denaturation of HSAff in H_2_O with and without ligands such as fatty acid (octanoic acid), diazepam and tolbutamide measured at 190 nm for 100 repeated consecutive Synchrotron radiation circular dichroism (SRCD) spectra. (**c**) Rate of UV protein denaturation of antibody Mab-1 in different formulation agents for 30 repeated consecutive Synchrotron radiation circular dichroism (SRCD) spectra. The graph is reported in percentage of Protein folding damage calculated by dividing the ellipticity at 205 nm of the protein-ligand complex by that of the protein alone and multiplied by 100

Protein-Ligand binding interactions can be assessed by a variety of techniques such as Isothermal Calorimetry (Isothermal Calorimetry (ITC))Isothermal Calorimetry (ITC), Surface Plasmon Resonance (SPR), Fluorescence and Circular Dichroism (Circular dichroism (CD))Circular dichroism (CD). Each technique has its own strengths and weaknesses. ITC can often be inconclusive when the thermal effects associated with the displacement of the bound solvent molecules by the ligand are rather small. SPR requires one of the components to be immobilized, which can lead to false positive or false negative results when compared to non-immobilized conditions. Fluorescence requires the presence of a fluorophore that does not absorb and emit light at the same wavelength for the ligand and/or protein (Tryptophan and Tyrosine residues). Circular dichroism (CD) relies on detectable spectral differences between the observed spectrum of the protein-ligand mixture at (x:y) molar ratio and the calculated sum of the spectra of the protein at (x) molar ratio and ligand at (y) molar ratio. For achiral ligands, the comparison is facilitated by the fact that the ligand chromophore will acquire an induced Circular dichroism (CD) upon binding of a protein site that is chiral. In this case, the detection of any induced Circular dichroism (CD), which is any spectral difference from that of the protein alone, is unambiguously indicative of the presence of a bound species, as the free achiral form is devoid of any Circular dichroism (CD) from its UV absorbing chromophore (Siligardi and Hussain 1998; Hussain et al. 2012b; Siligardi et al. 2002; Martin et al. 2011). However, for ligands with weak or with no UV chromophore groups, such as sugars, lipids and irregular peptides, the lack of any Circular dichroism (CD) spectral change upon ligand addition does not necessarily signify that there are no binding interactions. The ligand might bind far from the aromatic side-chains of the protein, without causing any detectable secondary structure conformational change. For these cases, the protein UV denaturation assay using the B23 beamline was developed and is now part of the facilities available at the Diamond B23 beamline (Hussain et al. 2012a, b; Jávorfi et al. 2010). High UV photon flux at B23 was used to qualitatively distinguish the binding of ligands to proteins, such as non-steroidal anti-inflammatory drugs to serum albumin, though not a membrane protein is a transporter (Fig. 4.2) (Hussain et al. 2012a). UV denaturation can also be used to discriminate the effects of different excipients, such as Detergents, salts, and buffers on peptide hormones such as vasoactive peptide VIP, which binds to a G protein coupled receptors (GPCRs) (Longo et al. 2015) and antibodies (such as cetuximab which is an inhibitor of EGFR, a transmembrane protein) on the effect of formulating agents (Longo et al. 2015; Siligardi and Hussain 2015). These measurements are easy to carry out, as it only requires repeated scanning of samples and a plot of the denaturation decay, which will provide differential dynamics properties of the system.

Binding studies using the Circular dichroism (CD) titration method are even more challenging for membrane proteins than they are for soluble proteins. A common limitation encountered with membrane proteins is that they are often expressed in rather small quantities. At the B23 beamline, the highly collimated incident micro-beam (0.3 × 0.5 mm) is enabling the use of a small aperture cuvette cells of low volume capacity (from few μl to 25 μl for 0.01 cm to 1 cm pathlengths) to measure the Synchrotron radiation circular dichroism (SRCD) of precious and scarce membrane proteins.

**Fig. 4.3:**
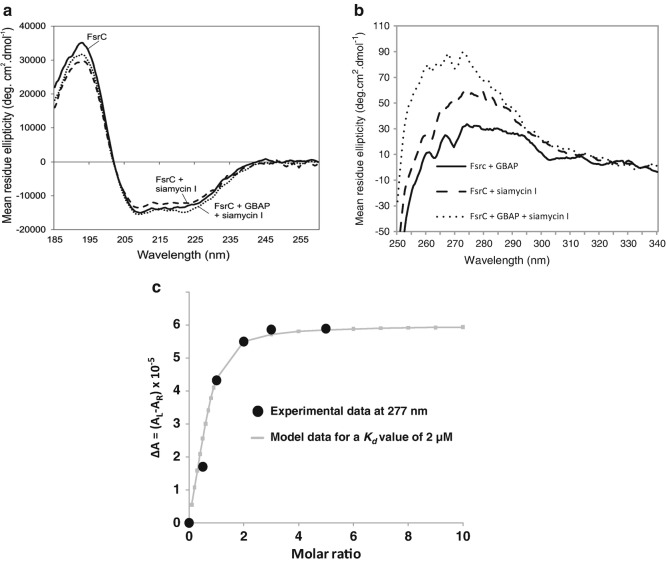
Binding interaction properties of FsrC membrane protein determined using the B23 beamline for synchrotron radiation circular dichroism (Synchrotron radiation circular dichroism (SRCD)) (a, b, c figures redrawn from Patching et al. 2012 and Phillips-Jones et al. 2013). (**a**) Far-UV Synchrotron radiation circular dichroism (SRCD) spectra of FsrC membrane protein with (*dashed*) and without (*solid*) GBAP ligand Patching et al. 2012. (**b**) Near-UVSynchrotron radiation circular dichroism (SRCD) spectra: (*top*) FsrC with (*dashed*) and without (*solid*) GBAP ligand. The insert is the fitting of the GBAP Synchrotron radiation circular dichroism (SRCD) titration into FsrC protein using a non-linear regression analysis. The Kd calculated from Circular dichroism (CD) data was 2 μM, (*bottom*) FsrC with (*dashed*) and without (*solid*) Siamycin I ligand. (**c**) Near-UV Synchrotron radiation circular dichroism (SRCD) spectra of (FsrC + GBAP) (*thick black*), (FsrC + Siamycin I) (*dashed*) and (FsrC + GBAP + Siamycin I) (*thin black*). The unique micro-collimated beam of Diamond’s B23 beamline enabled the measurements to be carried out using a small volume capacity cell (50–100 μl) of 1 cm pathlength otherwise unattainable with bench-top Circular dichroism (CD) instruments

FsrC, a membrane protein histidine kinase, showed very little conformational changes in secondary structure upon addition of ligand Gelatinase Biosynthesis-Activating Pheromone (GBAP). However, the local tertiary structure of FsrC showed significant changes when GBAP was added, indicating the involvement of aromatic residues binding to GBAP, see Fig. 4.3. Qualitative studies showed that the affinity of the ligand GBAP to FsrC was 2 μM, monitored at a single wavelength of 277 nm. Further competitive binding studies with another ligand, siamycin I, showed that siamycin I did not compete with GBAP and that aromatic side-chain residues should be involved in the interaction with the ligand (Patching et al. 2012; Phillips-Jones et al. 2013).

**Fig. 4.4:**
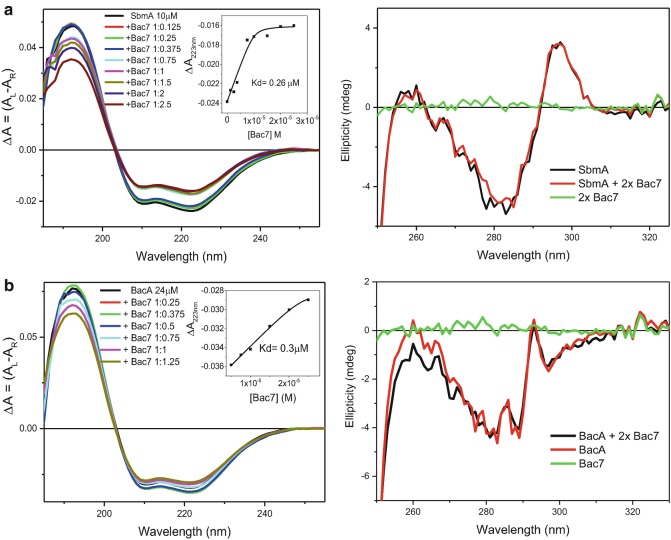
(**a**) Synchrotron radiation circular dichroism (SRCD) spectra of SbmA with and without Bac7 in the Far-UV (*left*) and Near-UV (*right*) regions. The insert in the Far-UV region shows the determination of the dissociation constant Kd calculated to be 0.28 μM by fitting the Circular dichroism (CD) data at 223 nm versus Bac7 concentration using the non-linear regression analysis (Siligardi et al. 2002) of CDApps (Hussain et al. 2015). (**b**) Synchrotron radiation circular dichroism (SRCD) spectra of BacA with and without Bac7 in the Far-UV (*left*) and Near-UV (*right*) regions. The insert in the Far-UV region spectra shows the determination of the dissociation constant Kd calculated to be 0.30 μM by fitting the Circular dichroism (CD) data at 223 nm versus Bac7 concentration using the non-linear regression analysis of CDApps

SbmA, a bacterial inner membrane protein of Gram-negative bacteria is involved in the transport within the cell of prokaryotic and eukaryotic antimicrobial peptides and glycopeptides, as well as of peptide-nucleic acid (PNA) oligomers. A SbmA homolog, BacA, is required for the development of *Sinorhizobium meliloti* bacteroids within plant cells and favours chronic infections with *Brucella abortus* and *Mycobacterium tuberculosis* in mice. A Synchrotron radiation circular dichroism (SRCD) spectroscopic study provided evidence that SbmA and BacA interact *in vitro* with Bac7 (1–35), a proline rich peptide. Bac7 was titrated at various molar ratios to SbmA and BacA in both the Far-UV (180–260 nm) and Near-UV (250–330 nm) regions. In the Far-UV region, significant changes of secondary structure were observed upon addition of Bac7 to SbmA (see Fig. 4.4a and Table 4.1) and BacA (Fig. 4.4b) indicating binding interactions. In the Near-UV region (Runti et al. 2013), characteristic of the local tertiary structure of aromatic side-chain residues (Tryptophan, Tyrosine and Phenylalanine) (Siligardi et al. 1991; Siligardi and Hussain 1998; Hussain et al. 2012b; Gaspar et al. 2011) no significant changes were observed, which suggests that no aromatic residues were involved at close range (radius of 6 Å) in the interface between ligand and protein. The Synchrotron radiation circular dichroism (SRCD) spectra were converted to ΔA = (A_L_−A_R_) units from millidegree units and the plot of ΔA intensities at 223 nm versus the concentrations of Bac7 used in the titration was analysed with a non-linear regression method (Siligardi et al. 2002) to quantitatively determine the dissociation constant using CDApps software (Hussain et al. 2015). The results showed that the peptide had similarly high binding Binding affinity to SbmA (Kd of 0.26 μM) and BacA (Kd of 0.3 μM).

**Table 4.1:** Protein Secondary Structure content of SbmA and BacA membrane proteins with and without addition of Bac7 ligand up to 1:35 molar ratios calculated from Synchrotron radiation circular dichroism (SRCD) data using CONTINLL algorithm (Sreerama and Woody 2004) with SMP50 reference data set of 37 soluble proteins and 13 membrane proteins applied through CDApps beamline software (Hussain et al. 2015)

Protein secondary structure elements (SSE)	SbmA	SbmA + Bac7 [1–35]	BacA	BacA + Bac7 [1–35]
H1, α-helix	0.41	0.32	0.41	0.46
H2, distorted α-helix	0.19	0.17	0.19	0.19
S1, β-strand	0.04	0.09	0.06	0.03
S2, distorted β-strand	0.04	0.04	0.03	0.02
T, turn	0.12	0.10	0.12	0.10
U, unordered	0.21	0.28	0.20	0.20
Spectral fit SD	0.06	0.04	0.06	0.04

Redrawn from Runti et al. 2013

**Fig. 4.5:**
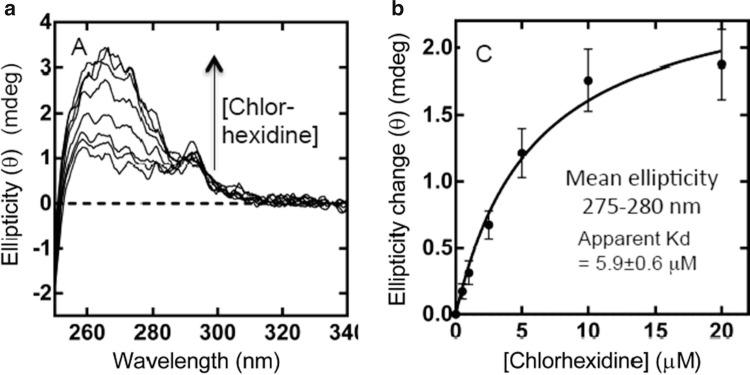
Synchrotron radiation circular dichroism (SRCD) titration of chlorhexidine into 20 μM purified wild-type AceI protein. (**a**) Near-UVSynchrotron radiation circular dichroism (SRCD) spectra of AceI protein with and without chlorhexidine. The arrow indicated the spectra with increased concentration of chlorhexidine. (**b**) Plot of ellipticity (θ) intensity versus chlorhexidine concentration. The fitting curve (*solid line*) of the experimental data (*solid black circles*) was calculated using the non-linear regression analysis with a Kd of 5.9 μM (Siligardi et al. 2002)

For the membrane protein Ace1 (actinobacter chlorhexidine efflux transport system 1), no significant conformational changes in the Far-UV region (180–250 nm) were observed in the presence of an achiral chlorhexidine antibacterial drug (Hassan et al. 2013). However, in the Near-UV region, the Synchrotron radiation circular dichroism (SRCD) titration unambiguously showed the induced Circular dichroism (CD) of the bound chorhexidine to the AceI membrane protein (Fig. 4.5a). The non-linear regression analysis of the Synchrotron radiation circular dichroism (SRCD) data revealed adissociation constant (K_d_) of 6 μM (Fig. 4.5b). In this example the binding was demonstrated by the increased induced Circular dichroism (CD) at about 270 nm, which reach a plateau upon saturation. The Ligand binding did not perturb the content of the secondary structure of Ace1, as illustrated by the lack of conformational changes in the Far-UVSynchrotron radiation circular dichroism (SRCD) spectrum.

## Protein Stability

Throughout the course of protein production, different batches of proteins are expressed and different protocols or procedures for purification are used. These variables can have an effect on the integrity of the proteins. This is especially relevant to membrane proteins that have to be solubilised in different Detergents for stabilisation and for Crystallisation with potential ligand molecules.

**Fig. 4.6:**
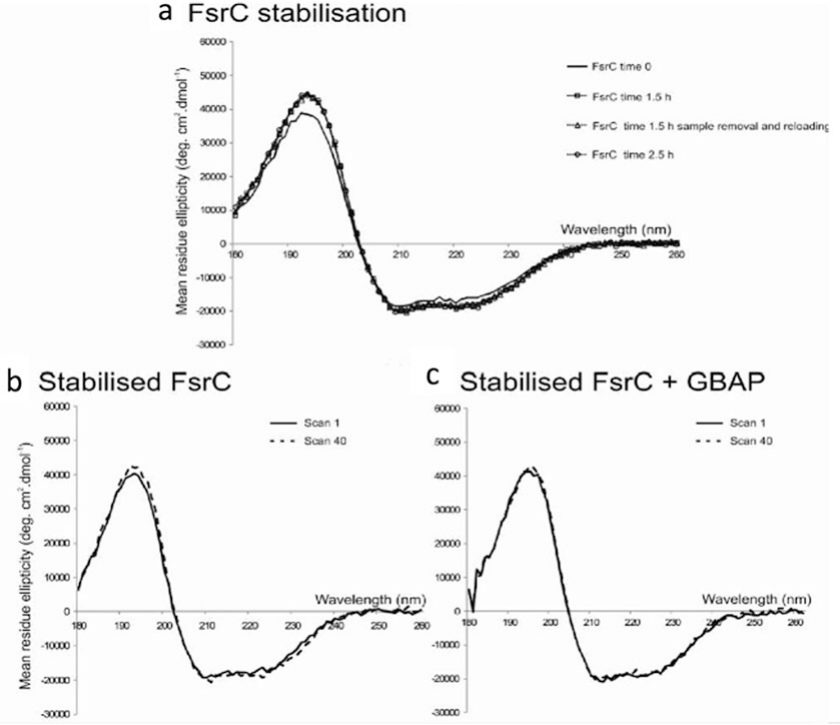
FsrC stability determined by Synchrotron radiation circular dichroism (SRCD) spectroscopy. (**a**) Far UV Synchrotron radiation circular dichroism (SRCD) spectra of purified FsrC (6 μM) at 0 h (*solid black line*), 1.5 h (*dashed line, unfilled square*), and 2.5 hr (*dashed line, unfilled circle*) following sample preparation in 10 mM sodium phosphate pH 7.5 containing 0.02 % in n-Dodecyl β-D-maltoside (DDM) at 20 °C. Repeated Synchrotron radiation circular dichroism (SRCD) spectra after 1.5 h involving sample removal and reloading were also included (*dashed line, unfilled triangle*). Synchrotron radiation circular dichroism (SRCD) spectra of stabilised FsrC before and after exposure to far UV radiation illustrated in the absence (**b**) and in the presence (**c**) of twofold GBAP ligand. Spectrum 1, immediately after stabilisation (solid line); and spectrum 40, following 100 min exposure to light radiation in the 190–260 nm spectral region during 39 repeated consecutive scans (*dashed line*)

The example of the FsrC protein (Patching et al. 2012) showed that it required 1.5 h of incubation time for stabilisation, which was determined by measuring consecutive repeated scans of about 3 min each in the 260–180 nm region until the dominating Alpha helical spectral feature stopped increasing at about 30 scans, see Fig. 4.6. It was essential to know the specific equilibration time for FsrC, and more generally, the equilibration time for membrane proteins in each specific Detergents, when assessing Ligand binding interactions, as otherwise it might result in ambiguous results. Interestingly, for FsrC the addition of ligand peptide GBAP appeared to stabilize the protein rather quickly (Fig. 4.6). The sugar transport protein GalP was also monitored for stability over time using the repeated scan method using high UV photon flux (Kalverda et al. 2014) showing that GalP is stable over repeated scans using a smaller bandwidth of 1.1 nm.

## Temperature Denaturation

Temperature denaturation studies on proteins are routinely used to determine the thermodynamic properties of wild type proteins, their mutants, and the effect of Ligand binding interactions or that of excipients as stabilisers to withstand long protein storage and transport conditions.

**Fig. 4.7:**
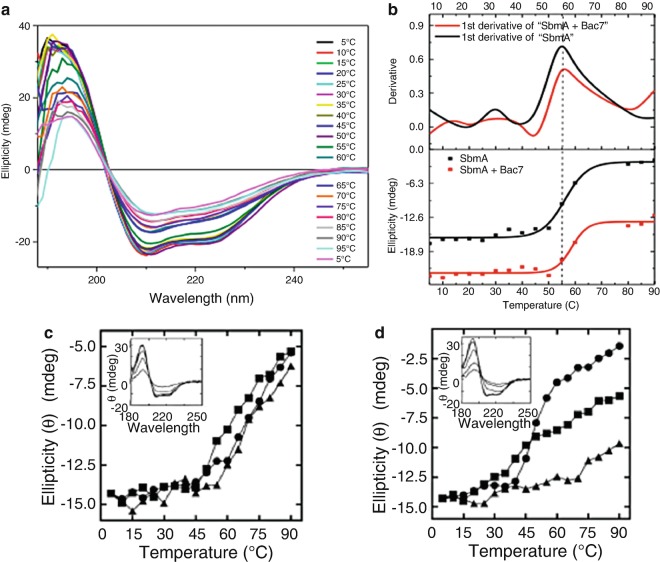
(**a**) Synchrotron radiation circular dichroism (SRCD) spectra of SbmA + Bac7 [1:5] as a function of temperature (Runti et al. 2013). (**b**) Thermal denaturation of SbmA with and without Bac7 ligand using Synchrotron radiation circular dichroism (SRCD) spectroscopy. The upper part of the figure is the determination of the melting temperature Tm by 1st derivative method whilst the lower part is by the Boltzman equation. The lower part is also used to represent the thermal denaturation property or the thermal stability of the protein (Runti et al. 2013). (**c**) Thermal stability of wild type Ace1 protein (33 μM) with and without chlorhexidine by Synchrotron radiation circular dichroism (SRCD) (redrawn from Hassan et al. 2013). (**d**) Thermal stability of mutant Ace1 protein E15Q (33 μM) with and without chlorhexidine by Synchrotron radiation circular dichroism (SRCD) (redrawn from Hassan et al. 2013). For both (**c**) and (**d**), the ellipticity at 209 nm is shown for protein only (●), protein plus 100 μM chlorhexidine (■; 1:3 molar ratio) and protein plus 500 μM chlorhexidine (▲; 1:15 molar ratio) at increasing temperature. Insets show the characteristic α-Alpha helical protein Far-UV spectrum of the respective proteins at increased temperature in the absence of chlorhexidine (redrawn from Hassan et al. 2013)

However, for the SbmA protein (described in Sect. 4.4) the thermal behaviour was not affected by the addition of peptide Bac-7 (Fig. 4.7a), even though conformational changes by Synchrotron radiation circular dichroism (SRCD) were observed in the Far-UV region (Fig. 4.4) (Runti et al. 2013). The Synchrotron radiation circular dichroism (SRCD) titration of Bac7 with SbmA indicated that the molecular interaction was accompanied by an 11 % decrease in Alpha helical content for either SbmA or Bac7 (Table 4.1). The thermal studies, however, showed no significant differences between the melting temperature (T_m_ = 55 °C) of SbmA and the melting temperature of a mixture of SbmA and Bac7 (Fig 4.7).

In the temperature denaturation study of the Ace1 protein (Kalverda et al. 2014) the protein melting temperatures were measured by ramping the temperature from 5 to 90 °C while monitoring the Far-UVCircular dichroism (CD) spectrum, particularly at 209 nm or 222 nm. The wild-type protein was observed to be rather stable at temperatures below 60 °C. However, mutant E50Q Ace1, known to impair resistance to chlorhexidine, showed thermal denaturation at 40 °C. Chlorhexidine binding greatly increased the thermal stability of the E50Q mutant protein in a dose-dependent manner. Importantly, these experiments yielded evidence, albeit indirect, that the Ace1 protein was itself involved in binding chlorhexidine as an integral feature of the resistance mechanism.

**Fig. 4.8:**
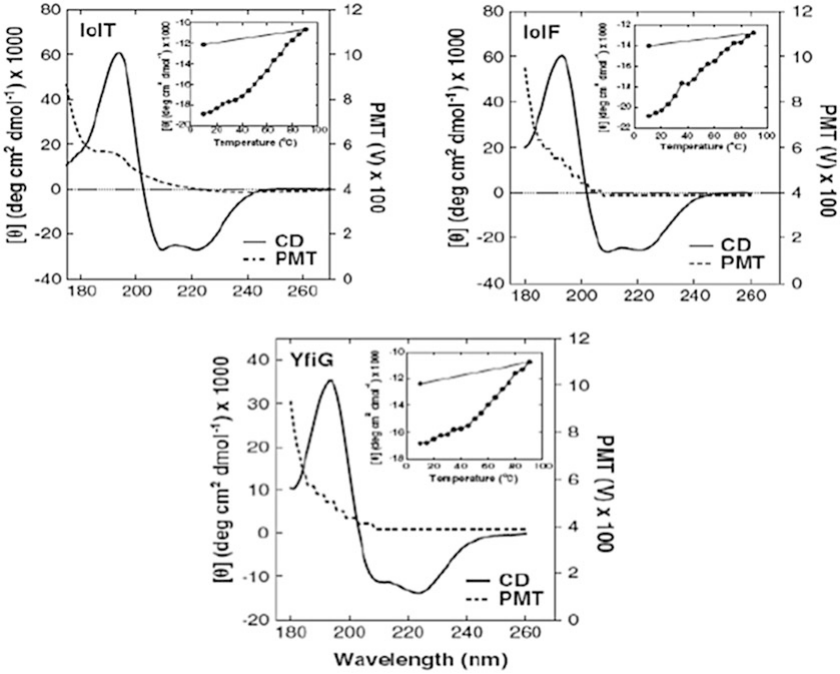
Analysis of secondary structure and thermal denaturation profiles in the purified proteins. Synchrotron radiation circular dichroism (Synchrotron radiation circular dichroism (SRCD)) spectra at 20 °C in the Far-UV region are shown for the purified IolT(His_6_), IolF(His_6_) and YfiG(His_6_) proteins at a concentration of 20 μM in a buffer containing 0.05 % DDM and 10 mM potassium phosphate (pH 7.6) using a 0.2-mm pathlength cell and acquiring spectra with an interval time of 0.5 nm. The spectra are buffer-subtracted and are the average of four scans. The plots show the Circular dichroism (CD) spectrum (Circular dichroism (CD)) and the high tension (HT) voltage values. Inset are thermal stability profiles at a wavelength of 222 nm from Circular dichroism (CD) spectra recorded over a range of temperatures (10–90 °C and then back to 10 °C) for each of the proteins at a concentration of 0.1 mg/ml (redrawn from Bettaney et al. 2013)

In a study by Bettaney et al. (2013), the Synchrotron radiation circular dichroism (SRCD) spectra of three inositol membrane transport proteins (IolF, IolT, and YfiG) measured with the Diamond B23 beamline showed a cut-off below 180 nm, indicated by the voltage of the photomultiplier tube (PMT) detector exceeding 600 V (Fig. 4.8). It is essential for accurate secondary structure estimations that the positive Circular dichroism (CD) bands at about 190–195 nm of the α-Alpha helical conformation are measured with the lowest level of noise possible, which can be readily achieved with synchrotron Circular dichroism (CD) beamlines. Repeated scans can also improve the signal-to-noise-ratio, but at an increased overall time (the noise is reduced by the root square of the number of scans). Following successful confirmation of secondary structural integrity and composition, the specificity of IolF, IolT, and YfiG for a large variety of inositols and sugars of D and L configurationn was determined by measuring the cellular uptake of radiolabelled 3H-myo-inositol in the presence of unlabelled competing compounds (Bettaney et al. 2013) as well as differences in thermodynamic properties, which could give indications of any concerns for Crystallisation trials.

## Conformational Analysis and HT-CD Screening of Protein Crystallisation Buffers

The assessment of Protein folding in solution is particularly important because conformational changes among a wild type protein and its mutants/constructs are likely to affect the protein activity and stability. Protein folding plays a crucial role in the function of a protein and its characterisation is a prerequisite for the understanding of the mechanism and consequences of sequence amino acid point mutation that could trigger protein misfolding for insulin, α-synuclein, lysozyme, and transthyrethin to name but a few cases with serious health implications (Blancas-Mejía and Ramirez-Alvarado. 2013; Ruzza et al. 2014, 2015; Marchiani et al. 2013).

A recent example regarding the importance of Protein folding characterisation (including different constructs of the same protein) by Synchrotron radiation circular dichroism (SRCD) is the case of the membrane protein GDP-mannose-dependent mannosyltransferase WbdD. In this example, the accurate determination of the α-Alpha helical content of the protein was crucial as a molecular ruler to regulate O-antigen chain length in lipopolysaccharide of *E coli* 09A (Hagelueken et al. 2015).

**Fig. 4.9:**
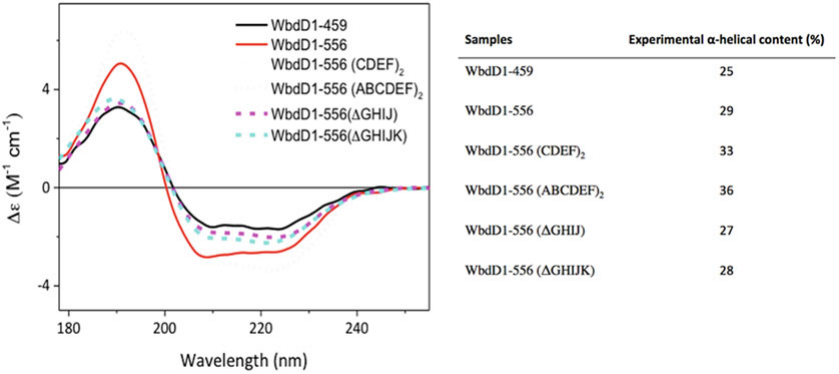
Circular dichroism (CD) spectra of different WbD constructs and their secondary structure content estimation using CONTINLL (Sreerama and Woody 2004) of Circular dichroism (CD) Apps beamline software (Hussain et al. 2015). On the right is shown the percentage of α-Alpha helical content calculated from Synchrotron radiation circular dichroism (SRCD) data using CONTINLL algorithm in CDApps beamline software

WbdD is a membrane-associated protein and its interaction with WbdA (another GDP-mannose-dependent mannosyltransferase) is essential for the ability of the soluble polymerase to act on the membrane-embedded undecaprenyl lipid–linked acceptor (Clarke et al. 2009). The authors investigated two insertion and two deletion variants of WbdD1–556 to gain some structural insight into the effect of the changes in the coiled-coil region. Multi-techniques such as the crystal structure of WbdD1-556 showing the coiled-coil region, molecular modelling and small angle X-ray scatterings (SAXS) models were coupled to elucidate the 3D structure. The study was further expanded with a bioassay of different constructs, giving various lengths of coupled lipopolysaccharides with Circular dichroism (CD) spectroscopy. Circular dichroism analysis of WbdD1–459 and WbdD1–556 using the CONTINLL algorithm (Sreerama and Woody 2004; Hussain et al. 2015) on spectra collected on B23 at Diamond confirmed a detectable increase in α-Alpha helical content, as was expected by the addition of a coiled-coil region (Fig. 4.9). This multi-technique study helps to piece together information gathered and complements the limitations of each of the individual techniques into a final and more complete understanding of the role of the coiled-coil region of the protein as a molecular ruler.

The high photon flux of the B23 beamline has been successfully used to develop a protein UV-denaturation assay that can discriminate the relative stability of different types of Protein folding and also to qualitatively determine Ligand binding interactions (Hussain et al. 2012a, b; Jávorfi et al. 2010; Longo et al. 2014, 2015). The latter application has been very useful to study ligands with weak or no UV chromophores that would otherwise be difficult or impossible to monitor by conventional benchtop Circular dichroism (CD) spectrometer.

**Fig. 4.10:**
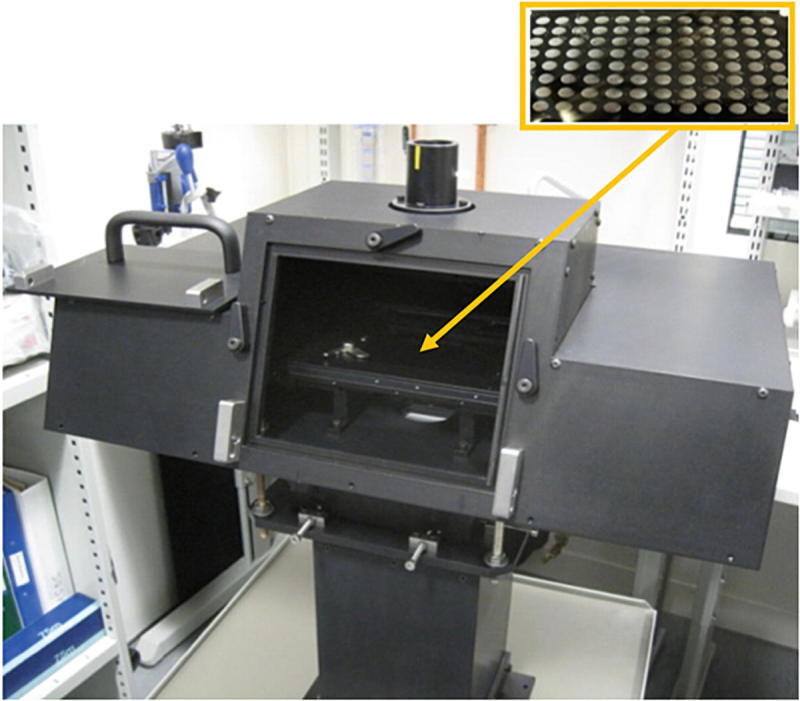
Vertical sample compartment of Diamond B23 beamline module A. The chamber enables the Synchrotron radiation circular dichroism (SRCD) measurements of horizontally positioned samples at respect to the incident monochromatic light. It has been designed to accommodate the 96- and 384-well multiplates made of fused quartz (Suprasil, Hellma). The central insert shows where the enlarged 96-well multiplate is located inside the chamber (*yellow arrow*)

The conformation of peptides and proteins is known to be affected by environmental conditions such as buffer composition, pH, salt concentration, Detergents, metal ions and precipitants. The latter is widely used in crystallography to enhance crystallisation. However, the use of Circular dichroism (CD) spectroscopy for such characterisations would be rather laborious and time consuming as this would normally be carried out by measuring the samples in a single cuvette cell, one by one. The unique highly collimated incident micro-beam (from 0.3 mm^2^ to 1.5 mm^2^ cross section) of the B23 has recently been exploited for the use of 96- or 384-well plates, to allow for high throughput Circular dichroism (CD) (HT-CD) (Fig. 4.10) screening to characterise Protein folding in crystallisation conditions and in protein-drug binding interactions (Siligardi and Hussain 2015).

**Fig. 4.11:**
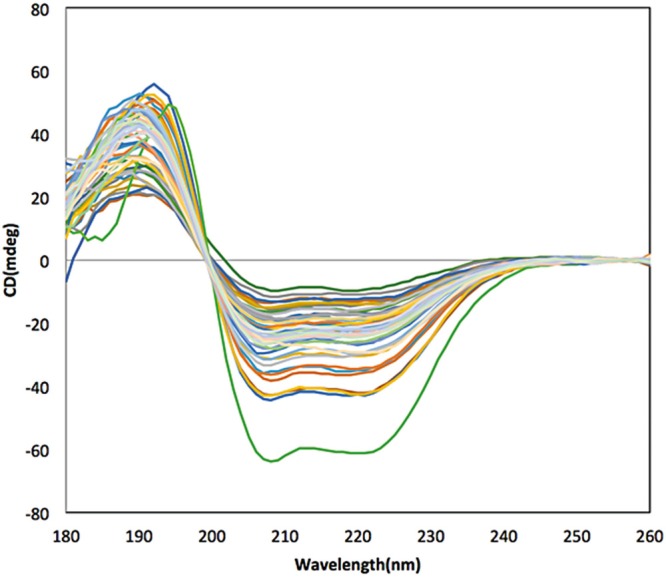
Synchrotron radiation circular dichroism (SRCD) spectra of myoglobin dissolved in 48 distinct buffers of MemGold2 for membrane protein crystallisation

**Fig. 4.12:**
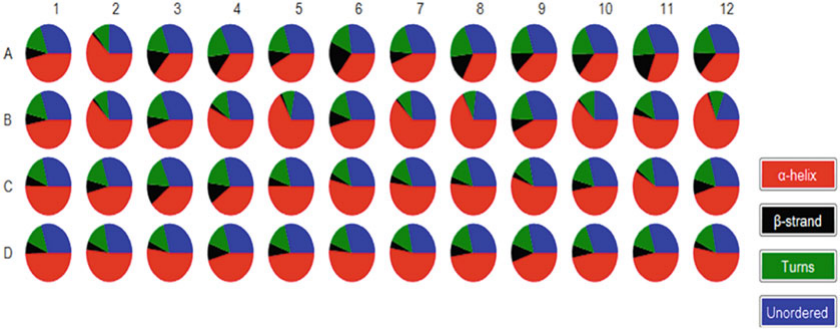
Secondary structure content of myoglobin dissolved in the 48 crystallographic solvent conditions of the MemGold2 multiplate (well coordinates A1-12, B1-12, C1-12 and D1-12). The pie chart was prepared using B23 CDApps software (Hussain et al. 2015). Although the α-helix content is the dominating element of secondary structure, significant different a-Alpha helical contents were induced by some of the MemGold2 solvent conditions. At first glance less helical content can be observed in A3, A4, A6, A8-12, C3, C4, D9 (12 out of 48 or 25 %) whilst more helical in B3, B4, B7, B8, B10, B12 and C11 (6 out of 48 or 12.5 %)

Myoglobin, a highly α-Alpha helical protein, was investigated by Synchrotron radiation circular dichroism (SRCD) spectroscopy dissolved in a selection of 48 conditions from the MemGold2 crystallisation screen (Molecular Dimensions) that is widely used for membrane protein crystallization. The corresponding 48 Synchrotron radiation circular dichroism (SRCD) spectra (Fig. 4.11) showed significant conformational differences that could be attributed to electrolyte concentration and pH. It is important to note that despite myoglobin being a highly α-Alpha helical soluble protein its conformation can be perturbed by the membrane protein crystallisation buffer MemGold2. This can be readily illustrated in the pie chart of the secondary structure content determined by Synchrotron radiation circular dichroism (SRCD) spectroscopy using CONTINLL of the Synchrotron radiation circular dichroism (SRCD) spectra of the 96-well multiplate (Fig. 4.12).

Most membrane proteins are highly helical and it is not inconceivable that they too might behave similarly to myoglobin under various buffer conditions. This is consistent with the fact that membrane proteins only crystallise in certain buffer conditions (Privé 2007; O’Malley 2011) but not in others. This raises the question whether proteins that do not crystallise do not do so because they have different conformations that do not promote association or that their thermodynamic properties are significantly different, inhibiting crystal formation.

The availability of HT-CD facility at the B23 beamline opens up the possibility of finding cross-correlations between buffer conditions and protein conformation, identifying possible buffer targets that could promote crystallisation for further 3D structural determination by X-ray crystallography.

## Conclusion

The work described in this chapter highlights the use of Circular dichroism (CD) and Synchrotron radiation circular dichroism (SRCD) spectroscopy for studies of membrane proteins, particularly when a wide spectral range from Far-UV to Near-UV region (180–350 nm) is achieved for both protein and ligand. This approach enables the determination of the stability and content of the Protein Secondary Structure, the qualitative and quantitative assessment of Ligand binding interactions and the characterisation of the solvent (Detergents) conditions to optimise protein stability and binding properties. It has been successful in the identification of drugs that despite similar activity revealed different thermodynamics properties when bound to various protein constructs.

The amide bond of the protein backbone structure gives information about the Protein Secondary Structure while the aromatic side-chains of the tryptophan, tyrosine and phenylalanine amino acid residues provide details about their local tertiary structure, as such, these are ideal as molecular probes for Ligand binding interactions. The chromophore of the ligand can be used to unambiguously determine Ligand binding in both far- and Near-UV regions and in particular whether protein conformational changes are occurring upon Ligand binding. Further development in high throughput allows the screening of crystallization buffers and the potential screening of ligands in drug discovery with membrane proteins acting as host or target receptor. This broad approach of Circular dichroism (CD) spectroscopy in solution for membrane proteins is advantageous. Synchrotron radiation circular dichroism (SRCD) in particular provides wavelength extension in the vacuum UV region and higher photon flux, which enables more penetrating measurements with less transparent media (NaCl content up to 500 mM) and in small volume capacity cuvette cells that are unattainable using bench-top Circular dichroism (CD) instruments .
